# Continuity and Completeness of Electronic Health Record Data for Patients Treated With Oral Hypoglycemic Agents: Findings From Healthcare Delivery Systems in Taiwan

**DOI:** 10.3389/fphar.2022.845949

**Published:** 2022-04-04

**Authors:** Chien-Ning Hsu, Kelly Huang, Fang-Ju Lin, Huang-Tz Ou, Ling-Ya Huang, Hsiao-Ching Kuo, Chi-Chuan Wang, Sengwee Toh

**Affiliations:** ^1^ Department of Pharmacy, Kaohsiung Chang Gung Memorial Hospital, Kaohsiung, Taiwan; ^2^ School of Pharmacy, Kaohsiung Medical University, Kaohsiung, Taiwan; ^3^ School of Pharmacy, College of Medicine, National Taiwan University, Taipei, Taiwan; ^4^ Graduate Institute of Clinical Pharmacy, College of Medicine, National Taiwan University, Taipei, Taiwan; ^5^ Department of Pharmacy, National Taiwan University Hospital, Taipei, Taiwan; ^6^ Institute of Clinical Pharmacy and Pharmaceutical Sciences, National Cheng Kung University, Tainan, Taiwan; ^7^ Department of Population Medicine, Harvard Medical School and Harvard Pilgrim Health Care Institute, Boston, MA, United States

**Keywords:** electronic health records, healthcare system, data continuity, data completeness, data quality, Taiwan

## Abstract

**Objective:** To evaluate the continuity and completeness of electronic health record (EHR) data, and the concordance of select clinical outcomes and baseline comorbidities between EHR and linked claims data, from three healthcare delivery systems in Taiwan.

**Methods:** We identified oral hypoglycemic agent (OHA) users from the Integrated Medical Database of National Taiwan University Hospital (NTUH-iMD), which was linked to the National Health Insurance Research Database (NHIRD), from June 2011 to December 2016. A secondary evaluation involved two additional EHR databases. We created consecutive 90-day periods before and after the first recorded OHA prescription and defined patients as having continuous EHR data if there was at least one encounter or prescription in a 90-day interval. EHR data completeness was measured by dividing the number of encounters in the NTUH-iMD by the number of encounters in the NHIRD. We assessed the concordance between EHR and claims data on three clinical outcomes (cardiovascular events, nephropathy-related events, and heart failure admission). We used individual comorbidities that comprised the Charlson comorbidity index to examine the concordance of select baseline comorbidities between EHRs and claims.

**Results:** We identified 39,268 OHA users in the NTUH-iMD. Thirty-one percent (*n* = 12,296) of these users contributed to the analysis that examined data continuity during the 6-month baseline and 24-month follow-up period; 31% (*n* = 3,845) of the 12,296 users had continuous data during this 30-month period and EHR data completeness was 52%. The concordance of major cardiovascular events, nephropathy-related events, and heart failure admission was moderate, with the NTU-iMD capturing 49–55% of the outcome events recorded in the NHIRD. The concordance of comorbidities was considerably different between the NTUH-iMD and NHIRD, with an absolute standardized difference >0.1 for most comorbidities examined. Across the three EHR databases studied, 29–55% of the OHA users had continuous records during the 6-month baseline and 24-month follow-up period.

**Conclusion:** EHR data continuity and data completeness may be suboptimal. A thorough evaluation of data continuity and completeness is recommended before conducting clinical and translational research using EHR data in Taiwan.

## Introduction

Electronic health records (EHRs) are rich in clinical information and may serve as a good data source for research. However, as patients may seek care at different delivery systems for different conditions, the EHRs from a single medical center or delivery system may only contain a fraction of patients’ health data. Although EHR data reflect clinical decisions and practice patterns within the real-world setting, ensuring their completeness is critical for generating valid evidence (Weiskopf and Weng, 2013; Eichler et al., 2019). There is currently no standard definition of completeness of EHR data, but some researchers have measured it by comparing the number of encounters captured in the EHRs with the number of all encounters recorded by a more complete data source, e.g., a linked administrative claims database, which captures most or all medically attended events (Lin et al., 2018a). Studies conducted in the United States (Weiskopf and Weng, 2013; Lin et al., 2018b) and in Europe (Rinner et al., 2016) have evaluated the completeness of EHR data. A United States study found that only 16–27% of patient data are captured in a single EHR system. Compared to high EHR continuity, low EHR continuity can lead to up to an 18-fold greater misclassification of treatment exposure, outcome, or covariates that are used for confounding adjustment (Lin et al., 2018b).

To our knowledge, there had been no comprehensive evaluation of EHR data completeness in Taiwan. In Taiwan, the National Health Insurance (NHI) provides universal coverage, and patients are free to choose their healthcare providers and delivery systems for both primary and specialty care (Wu et al., 2010). Therefore, EHR data completeness may be different between Taiwan and Western countries. As the use of real-world data to support clinical or regulatory decision making has become a global movement, it is important to evaluate data completeness before conducting studies using EHR data in Taiwan.

This study evaluated EHR data completeness and continuity in patients prescribed an oral hypoglycemic agent (OHA) in three healthcare delivery systems in Taiwan. As in prior studies (Lin et al., 2018a; Lin et al., 2018b), we measured data completeness by comparing the number of encounters captured in the EHRs with the number of all encounters recorded in a linked claims database. We measured data continuity by examining whether patients had encounters recorded in successive 90-day intervals in the EHRs. We also assessed the concordance of select clinical outcomes and baseline comorbid conditions between the EHRs and the linked claims database.

## Materials and Methods

### Data Sources

The study included three EHR databases from three delivery systems: the Integrated Medical Database of National Taiwan University Hospital (NTUH-iMD), the Chang Gung Research Database (CGRD) from Chang Gung Memorial Hospitals (CGMHs), and the EHR database of National Cheng Kung University Hospital (NCKUH). The NTUH-iMD was used in the primary analysis, which evaluated EHR data continuity, data completeness, and EHR-claims data concordance. A secondary analysis was performed using all three EHR databases to assess EHR data continuity across different healthcare delivery systems. Data between June 2011 and December 2016 of these EHR databases were used for this evaluation.

We chose these three delivery systems because they reflect different levels of hospital care and geographical diversity. The NTUH (2,554 beds) and NCKUH (1,331 beds) are two medical centers located in the Northern and Southern metropolitan areas of Taiwan respectively. The CGMHs (9,584 beds) consist of two medical centers, two regional hospitals, and three district hospitals located in different cities in Taiwan. The three EHR systems cover 17% of the reimbursed healthcare services in Taiwan’s NHI program, which insures more than 99% of the 23 million population and includes all reimbursement-related data since 1997 (National Health Insurance Administration, 2020a).

The three EHR databases contain data on diagnosis, procedure, prescription and dispensing, and date of encounter. Diagnosis was recorded by International Classification of Diseases (ICD) Clinical Modification codes. The Ninth Revision of the ICD Clinical Modification codes (ICD-9-CM) were used before 2016, and the Tenth Revision of the ICD Clinical Modification codes (ICD-10-CM) were implemented in the beginning of 2016. Each hospital has its own coding systems for procedures and prescription medications, but all these codes can be mapped to standard codes for NHI reimbursement. We converted available EHR data in the three systems to a standard, common data model based on the Sentinel Common Data Model to facilitate multi-site data quality evaluation (Huang et al., 2021). Code mapping and data management were performed locally at each site. ICD codes and NHI reimbursement codes were used for data query across the three delivery systems. The EHR data used for this study were from subsets of the NTUH-iMD and the CGRD, which included patients with a diagnosis of hypertension (ICD-9-CM: 401.x; ICD-10-CM: I10.x), diabetes (ICD-9-CM: 250.x; ICD-10-CM: E11), dyslipidemia (ICD-9-CM: 272.x; ICD-10-CM: E78.x), or any cardiovascular disease (ICD-9-CM: 390.x-459.x; ICD-10-CM: I00.x-I99.x) between 2006 and 2017; the initial EHR data provided by the NCKUH were from a sample of patients with 1) at least one T2DM diagnosis (ICD-9-CM: 250.00, 250.02, 250.10, 250.12, 250.20, 250.22, 250.30, 250.32, 250.40, 250.42, 250.50, 250.52, 250.60, 250.62, 250.70, 250.72, 250.80, 250.82, 250.90, 250.92; ICD-10-CM: E11) and one OHA prescription, or 2) two outpatient T2DM diagnoses within 1 year between June 2011 to December 2016. Both outpatient and inpatient records were available in the NTUH-iMD and the CGRD, whereas only outpatient records were available in the NCKUH database at the time of this study.

In addition to the EHR databases, the nationwide claims database, National Health Insurance Research Database (NHIRD), was used to assess the completeness of NTUH-iMD. We considered the NHIRD as the reference for the evaluation of EHR data completeness because the NHI program in Taiwan provides universal coverage and comprehensive health services. Like other claims databases, the NHIRD does not capture out-of-pocket health services. However, OHAs and most care are fully covered by the NHI program, so misclassification due to out-of-pocket health services should be minimal in the present study. The NTUH-iMD was linked to the NHIRD by national identity numbers unique to each patient through a third party. NHIRD contains information on diagnosis, procedures, and prescription medication reimbursed by the NHI; up to three outpatient or five inpatient diagnosis codes per encounter are available in the NHIRD. We used the de-identified linked data from June 2011 to December 2016 to evaluate the completeness and concordance of the EHR data, using the linked claims data as the reference. The individual study protocols were approved by the institutional review boards of NTUH (201906066RINB), NCKUH (A-ER 108-097), and Chang Gung Medical Foundation (201900108B1).

### Study Cohort

The study cohort was individuals aged ≥20 years who were prescribed at least one OHA (see Supplementary Table S1 for codes). We chose these patients because they reflect mostly medically treated patients with type 2 diabetes mellitus (T2DM), who often have multiple comorbid conditions (American Diabetes Association, 2020; National Health Insurance Administration, 2020b). Healthcare services provided to T2DM patients with multiple comorbidities may be incompletely captured in the EHRs of a single delivery system.

### Electronic Health Record Data Continuity

**FIGURE 1:**
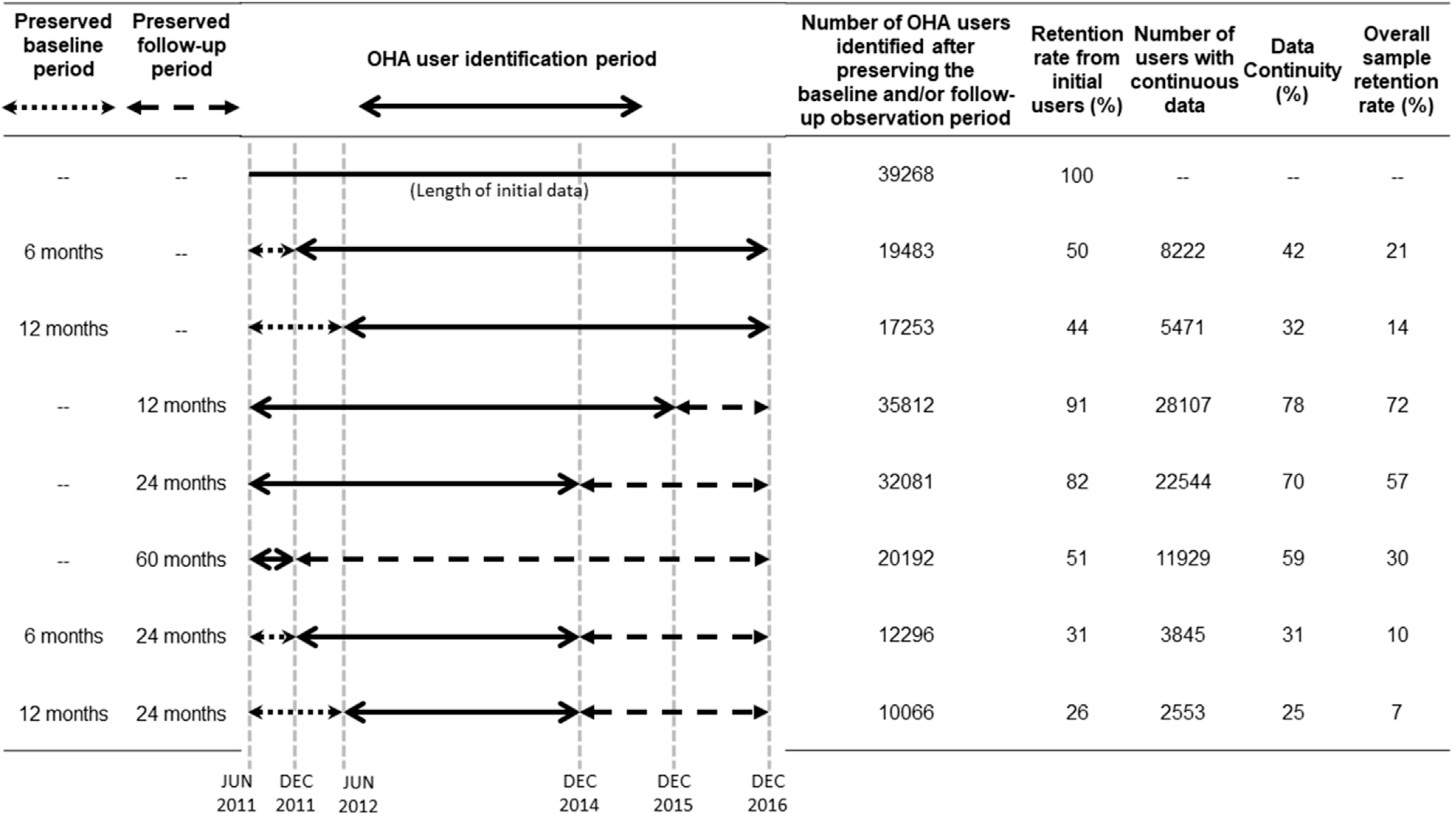
Cohort identification periods and numbers of eligible oral hypoglycemic agent users in the Integrated Medical Database of National Taiwan University Hospital. Solid lines represent the oral hypoglycemic agent (OHA) user identification periods; dotted lines represent the baseline periods of interest, and dashed lines represent the follow-up periods of interest. Baseline and follow-up periods were defined based on the first OHA prescription (i.e., the index date) observed in the OHA user identification period. Data continuity was assessed during the baseline and follow-up periods of interest. We defined patients as having continuous data if they had at least one encounter or prescription in the electronic health record database within a given 90-day interval.

We first assessed data continuity in the NTUH-iMD. Data continuity was examined in successive 90-day intervals before and after the index date, defined as the date of the first OHA prescription recorded in a given OHA user identification period (Figure 1). We defined patients as having continuous data if there was at least one encounter or prescription for any drug in a given 90-day interval. The 90-day interval was chosen because the NHI reimburses long-term prescriptions for up to 84 days (28 days’ supply × 3 refills) and requires patients to be re-evaluated for a new prescription. Therefore, although patients can fill their prescriptions at a hospital pharmacy or a community pharmacy, they must visit a healthcare provider for evaluation and review of their prescriptions approximately every 84 days.

We examined two pre-index (i.e., baseline) periods: 6 and 12 months, and three post-index (i.e., follow-up) periods: 12, 24, and 60 months. To obtain sufficient data to assess data continuity for different lengths of pre-index and post-index periods, the OHA user identification period, the data continuity assessment period, and the number of eligible OHA users varied by analysis. Figure 1 shows the relations among the OHA user identification period, baseline period, and follow-up period. A longer baseline or follow-up period would lead to a shorter OHA user identification period and fewer OHA users being identified. For example, patients with an index date of 1 December 2011 would not contribute to the analysis that examined data continuity during the 12-month baseline period because they would not have the opportunity to be followed for 1 year prior to the index date. We also measured sample retention, calculated as the proportion of OHA users with continuous data in different pre-specified baseline and follow-up periods among all OHA users identified in the study period (June 2011–December 2016).

### Electronic Health Record Data Completeness

We evaluated EHR data completeness by comparing the encounter records in the NTUH-iMD to the encounter records in the linked NHIRD among OHA users with at least 6 months of baseline and 24 months of follow-up data. Data completeness was measured by the mean proportion of encounters captured (MPEC), calculated by dividing the number of encounters in the NTUH-iMD by the number of encounters in the linked NHIRD (Lin et al., 2018a; Lin et al., 2020):

$$MPEC=\left(\frac{\text{Number}\;\text{of}\;\text{outpatient}\;{\text{encounters}}_{EHR}}{\text{Number}\;\text{of}\;\text{outpatient}\;{\text{encounters}}_{\mathrm{claims}}}+\frac{\text{Number}\;\text{of}\;{\text{admissions}}_{EHR}}{\text{Number}\;\text{of}\;{\text{admissions}}_{\mathrm{claims}}}\right).$$ (1)

Because NHI provides easy access and low out-of-pocket costs for medical care, people in Taiwan often seek for care even for minor diseases and symptoms, with an average of 15 visits per year (National Health Insurance Administration, 2021). As a result, the low MPEC could be affected by the amount of claims from local clinics for minor diseases and symptoms. Therefore, we performed a sensitivity analysis by excluding claims filed by local clinics and re-evaluated the data completeness with only claims filed by hospitals.

### Concordance of Select Clinical Outcomes

We evaluated the concordance of select outcomes between the NTUH-iMD and linked NHIRD in OHA users with continuous EHR data for at least 6 months before the index date and 24 months following the index date. We chose three major outcomes of diabetes: cardiovascular events, nephropathy-related events, and heart failure admission. Cardiovascular events were defined by a hospitalization or emergency department visit with a primary diagnosis of myocardial infarction (MI) or stroke. Nephropathy-related events were defined by a primary or secondary diagnosis of these events in any outpatient or inpatient records. The heart failure outcome was defined by hospitalization with a primary diagnosis of heart failure. All outcome events were assessed during the 24-month follow-up period. The diagnoses codes used to identify the outcomes are presented in Supplementary Table S2.

We selected these outcomes to represent a mix of events with different characteristics. We hypothesized that patients with MI or stroke, both of which are acute conditions, were more likely to have an emergency department or inpatient care in hospitals different than the ones they visit regularly for routine care. Since MI and stroke are potentially life-threatening events, patients are more likely to receive care from the nearest hospital when an event occurs, rather than being admitted to their routine care hospitals. Therefore, a low event capture rate was expected for cardiovascular events in the EHRs. Diabetes-associated nephropathy often develops gradually and is more likely to be diagnosed in outpatient visits; therefore, EHR data completeness was expected in patients with regular visits to the delivery system. Unlike acute MI and stroke, heart failure-related admission can be planned in Taiwan. We hypothesized that planned admissions were more likely to be captured by the EHRs studied.

Two indicators were calculated to examine the outcome concordance: EHR-claims outcome agreement and EHR outcome representativeness. We defined outcome agreement between the EHRs and the linked claims data as the number of patients whose outcome status were coded in the same way in both the EHRs and the linked claims database divided by the total number of patients who appeared in both databases. A patient defined as having the outcome in both the EHRs and the claims database, or not having the outcome in either database, would suggest high agreement. We defined outcome representativeness in the EHR database as the number of patients classified as having the outcome in both the EHRs and the claims database divided by the number of patients classified as having the outcome in the claims database. These two measurements reflected minimum outcome concordance as we did not require the event to appear on the same date between the EHRs and the linked claims database. The definitions of these two indicators are shown in Supplementary Table S3.

### Concordance of Select Baseline Comorbidities

We compared the baseline comorbidity profile during the 6-month baseline period between the NTUH-iMD and the linked NHIRD in OHA users with continuous EHR data for at least 6 months at baseline and 24 months following the index date. We examined the individual comorbidities that comprised the Charlson comorbidity index (Quan et al., 2005). Baseline comorbidities were identified by a primary or secondary diagnosis based on the ICD-9-CM or ICD-10-CM codes. We used an absolute standardized difference >0.1 to indicate a meaningful difference recorded between the EHRs and linked claims database (Lin et al., 2018b).

### Electronic Health Record Data Continuity in Different Healthcare Delivery Systems

To further understand whether EHR data continuity varied among healthcare delivery systems, a secondary analysis was conducted to compare the data continuity of three EHR databases from the NTUH, NCKUH, and CGMHs. To maintain consistent sample selection criteria across the health delivery systems, we first applied the sample selection criteria of the NCKUH dataset (i.e., at least one T2DM diagnosis and one OHA prescription, or two outpatient T2DM diagnoses within 1 year) on the NTUH-iMD and CGRD data and then selected OHA users for comparisons across the healthcare delivery systems.

## Results

### Electronic Health Record Data Continuity

We identified 39,268 OHA users in the NTUH-iMD between June 2011 and December 2016. As expected, the sample size decreased as we lengthened the baseline period due to the lower number of patients who could potentially be captured by the EHR system (Figure 1). We retained 50% and 44% of the original sample in the analyses that examined data continuity during the 6- and 12-month baseline period, respectively. Among these users, 42% and 32% had 6 and 12 months of continuous baseline data, respectively.

Similarly, the sample size decreased as we lengthened the follow-up period but the attrition was more modest: sample retention was 91%, 82%, and 51% in the analysis that examined 12-, 24-, and 60-month follow-up, respectively (Figure 1); the corresponding data continuity was 78%, 70%, and 59%, respectively.

Examining data continuity during the baseline and follow-up period simultaneously reduced the sample size significantly. For example, only 26% of the original sample of 39,268 OHA users contributed to the analysis that examined data continuity during the 12-month baseline and 24-month follow-up period; data continuity was 25% within this patient subset.

In addition, when considering baseline and follow-up data continuity together, we found that requiring longer baseline data continuity slightly improved the continuity of follow-up data but reduced the sample size considerably (Table 1). The proportion of patients with 24 months of continuous follow-up data was 70%, 74%, and 80% for OHA users with 0-, 6- and 12-month continuous baseline data requirement, respectively. However, the sample size dropped significantly from 22,544 (no baseline requirement) to 3,845 (6-month continuous baseline requirement) to 2,553 (12-month continuous baseline requirement).

**TABLE 1:** Data continuity during follow-up, by baseline continuity requirement, among oral hypoglycemic agent users who were eligible to be followed for at least 24 months in the Integrated Medical Database of National Taiwan University Hospital (*n* = 32,081).

		No baseline continuity requirement	At least 6 months of baseline continuity	At least 12 months of baseline continuity
		*n* = 32,081	*n* = 5,172	*n* = 3,205
		N (%)	N (%)	N (%)
With data continuity during follow-up (months)	3	29,972 (93)	4,961 (96)	3,099 (97)
	6	27,817 (87)	4,686 (91)	2,986 (93)
	9	26,474 (83)	4,493 (87)	2,895 (90)
	12	25,444 (79)	4,336 (84)	2,813 (88)
	15	24,653 (77)	4,211 (81)	2,758 (86)
	18	23,900 (75)	4,079 (79)	2,677 (84)
	21	23,216 (72)	3,956 (77)	2,610 (81)
	24	22,544 (70)	3,845 (74)	2,553 (80)

### Electronic Health Record Data Completeness

In the NTUH-iMD dataset that linked to the NHIRD claims data, the median MPEC was 42% (interquartile range, IQR, 20–68%) for OHA users with at least 6 months of continuous baseline data (*n* = 8,222), and 33% (IQR 21–49%) for OHA users with at least 24 months of continuous follow-up data (*n* = 22,544). For patients with continuous data for the entire 30 months (6-month baseline plus 24-month follow-up period, *n* = 3,845), the median MPEC was 41% (IQR 20–67%) for the 6-month baseline period, 48% (IQR 27–78%) for the 24-month follow-up period, and 52% (IQR 29–80%) for the entire 30-month period. The median MPEC increased to 50% in the 6-month baseline period (IQR 41–89%), 66% in the 24-month follow-up period (IQR 46–99%), and 71% (IQR 48–99%) in the entire 30-month period for OHA users when only hospital-level claims were counted.

### Concordance of Select Clinical Outcomes and Baseline Comorbidities

The outcome agreement and outcome representativeness of the NTUH-iMD compared to the linked NHIRD for select clinical outcomes are summarized in Table 2. Overall, outcome agreement between the NTUH-iMD and the linked NHIRD was high, but the NTUH-iMD only represented approximately 50% of the event recorded in the NHIRD. The prevalence of comorbidities recorded in the linked NHIRD was much higher than that recorded in the NTUH-iMD among OHA users with continuous 6-month baseline and 24-month follow-up data (Table 3). For OHA users with 6-month data continuity at baseline (*n* = 8,222), about 3% of them had a T2DM diagnosis in the NTUH-iMD during the 6-month baseline period, but approximately 68% of them had a T2DM diagnosis in the linked NHIRD.

**TABLE 2:** Outcomes captured in the Integrated Medical Database of National Taiwan University Hospital (NTUH-iMD) compared to the linked National Health Insurance Research Database (NHIRD) among oral hypoglycemic agent users with continuous 6-month baseline and 24-month follow-up data (*n* = 3,845).

		NHIRD		EHR-claims agreement	EHR outcome representativeness
		Yes	No		
a) Cardiovascular events					
NTUH-iMD	Yes	143	24	96%	51%
	No	140	3,538		
b) Nephropathy-related events					
NTUH-iMD	Yes	793	66	81%	55%
	No	648	2,338		
c) Heart failure admission					
NTUH-iMD	Yes	39	28	98%	49%
	No	40	3,738		

Outcome events were assessed during the 24-month follow-up period. We calculated EHR-claims agreement by dividing the number of patients whose outcome status were coded in the same way in both the EHRs and linked claims database by the total number of patients who appeared in both databases. A patient defined as having the outcome in both the EHRs and the claims database, or not having the outcome in either database, would suggest high agreement. We calculated EHR outcome representativeness by dividing the number of patients classified as having the outcome in both the EHRs and the claims database by the number of patients classified as having the outcome in the claims database. See Supplementary Table S3 for the definitions and calculations of EHR-claims agreement and EHR representativeness.

EHR, electronic health record.

**TABLE 3:** Comorbidities captured during the 6-month baseline period in the Integrated Medical Database of National Taiwan University Hospital (NTUH-iMD) and the linked National Health Insurance Research Database (NHIRD) among oral hypoglycemic agent users with continuous 6-month baseline and 24-month follow-up data (*n* = 3,845).

Condition^†^	NTUH-iMD	NHIRD	Absolute standardized difference
	N (%)	N (%)	
Myocardial infarction	<5 (<0.1)	114 (3.0)	0.24
Congestive heart failure	9 (0.2)	244 (6.4)	0.35
Peripheral vascular disease	5 (0.1)	109 (2.8)	0.23
Cerebrovascular disease	24 (0.6)	546 (14.2)	0.54
Dementia	5 (0.1)	112 (2.9)	0.23
Chronic pulmonary disease	13 (0.3)	431 (11.2)	0.48
Rheumatologic disease	<5 (<0.1)	46 (1.2)	0.15
Peptic ulcer	11 (0.3)	398 (10.4)	0.46
Liver disease	10 (0.3)	298 (7.8)	0.39
Mild liver disease	10 (0.3)	298 (7.8)	0.39
Moderate or severe liver disease	<5 (<0.1)	21 (0.6)	0.10
Diabetes	121 (3.2)	2108 (54.8)	1.39
Diabetes without chronic complication	119 (3.1)	2070 (53.8)	1.36
Diabetes with chronic complication	<5 (<0.1)	312 (8.1)	0.41
Hemiplegia or paraplegia	0 (0.0)	31 (0.8)	0.13
Renal disease	13 (0.3)	301 (7.8)	0.39
Malignancy	30 (0.8)	576 (15.0)	0.55
Metastatic solid tumor	<5 (<0.1)	48 (1.3)	0.15
AIDS/HIV	0 (0.0)	<5 (<0.1)	0.04

^†^Primary and secondary diagnoses from any visit.

### Electronic Health Record Data Continuity in Different Healthcare Delivery Systems

There were 38,134, 155,738, and 23,719 OHA users with at least one T2DM diagnosis identified in the NTUH-iMD, CGRD, and NCKUH EHR database, respectively, between June 2011 and December 2016 (Tables 4–6). Similar retention patterns of baseline data were observed between the NTUH-iMD and the CGRD. Less than half of the sample was retained in the NTUH-iMD and in the CGRD in the analysis that examined data continuity during the 6-month baseline period; among these patients, 41% had continuous baseline data in both NTUH-iMD and CGRD. In contrast, the NCKUH EHR database had a considerably higher retention compared to the other two EHR databases. Sample retention was 97% in the analysis that examined data continuity during the 6-month baseline period, and the corresponding data continuity was 64%.

**TABLE 4:** Sample attrition and data continuity among patients with type 2 diabetes mellitus in the Integrated Medical Database of National Taiwan University Hospital.

	Initial sample size, *n* = 38,134				
	Step 1: Number of patients eligible for analysis	Sample retention from initial sample to Step 1 (%)	Step 2: Number of patients with continuous baseline and/or follow-up data	Sample retention from Step 1 to Step 2 (%)	Overall sample retention from initial sample to Step 2 (%)
Baseline only					
At least 6-months baseline	18,649	49	7,621	41	20
At least 12-months baseline	16,291	43	5,015	31	13
Follow-up only					
At least 12-months follow-up	35,051	92	27,663	79	73
At least 24-months follow-up	31,538	83	22,297	71	58
At least 60-months follow-up	20,064	53	11,883	59	31
Baseline + Follow-up					
At least 6-months baseline + at least 24-months follow-up	11,873	31	3,697	31	10
At least 12-months baseline + at least 24-months follow-up	9,695	25	2,439	25	6

**TABLE 5:** Sample attrition and data continuity among patients with type 2 diabetes mellitus in the Chang Gung Research Database.

	Initial sample size, *n* = 155,738				
	Step 1: Number of patients eligible for analysis	Sample retention from initial sample to Step 1 (%)	Step 2: Number of patients with continuous baseline and/or follow-up data	Sample retention from Step 1 to Step 2 (%)	Overall sample retention from initial sample to Step 2 (%)
Baseline only					
At least 6-months baseline	74,964	48	30,838	41	20
At least 12-months baseline	65,858	42	19,150	29	12
Follow-up only					
At least 12-months follow-up	143,118	92	112,824	79	72
At least 24-months follow-up	129,267	83	90,620	70	58
At least 60-months follow-up	82,383	53	48,265	59	31
Baseline + Follow-up					
At least 6-months baseline + at least 24-months follow-up	48,493	31	14,058	29	9
At least 12-months baseline + at least 24-months follow-up	39,387	25	8,751	22	6

**TABLE 6:** Sample attrition and data continuity among patients with type 2 diabetes mellitus in the National Cheng Kung University Hospital electronic health record database.

	Initial sample size, *n* = 23,719				
	Step 1: Number of patients eligible for analysis	Sample retention from initial sample to Step 1 (%)	Step 2: Number of patients with continuous baseline and/or follow-up data	Sample retention from Step 1 to Step 2 (%)	Overall sample retention from initial sample to Step 2 (%)
Baseline only					
At least 6-months baseline	22,962	97	22,962	64	62
At least 12-months baseline	22,117	93	22,117	58	54
Follow-up only					
At least 12-months follow-up	21,486	91	21,486	82	74
At least 24-months follow-up	19,025	80	19,025	73	58
At least 60-months follow-up	11,668	49	11,668	62	30
Baseline + Follow-up					
At least 6-months baseline + at least 24-months follow-up	18,212	77	18,212	55	42
At least 12-months baseline + at least 24-months follow-up	17,304	73	17,304	54	39

Sample retention patterns were similar between the NTUH-iMD and the CGRD during follow-up. Sample retention was 83% in the analysis that examined 24-month follow-up continuity in both the NTUH-iMD and the CGRD; data continuity was 70% in these two databases. In the NCKUH EHR database, the proportion was 80% for sample retention and 73% for data continuity.

When considering baseline and follow-up data together, only 31% of the original sample contributed to the analysis that examined data continuity during the 6-month baseline and 24-month follow-up period in the NTUH-iMD and the CGRD, while 77% of the sample remained in the NCKUH EHR database. Data continuity for the entire 6-month baseline and 24-month follow-up period was approximately 30% in the NTUH-iMD and CGRD and 55% in the NCKUH EHR database. Extending the baseline from 6 to 12 months with a 24-month follow-up decreased the sample size without improving data continuity.

## Discussion

To the best of our knowledge, this is the first study in Taiwan that provides a comprehensive evaluation of EHR data from multiple large healthcare delivery systems. We adopted the perspective of an epidemiologist or real-world data scientist who would like to utilize the data for research and clinical investigation. In this study, less than half of the OHA users in the NTUH-iMD had continuous records in the 6-month period before their first recorded OHA prescription, whereas 78% and 70% had continuous 12 and 24 months of follow-up data, respectively. The median MPEC was 52% for patients with at least 6-month baseline and 24-month follow-up data in the NTUH-iMD. The NTUH-iMD only captured half of the cardiovascular events, nephropathy-related events, and heart failure admissions recorded in the linked NHIRD database. The concordance of important baseline comorbidities was also low. Therefore, the EHRs may not fully capture potentially important clinical outcomes or covariates, which may limit their applications in certain research studies. The fact that only 3% of OHA users had a diagnosis of T2DM in the NTUH-iMD (vs. 68% in the linked claims database) within 6 months before their first observed OHA prescription record also highlights the challenge of identifying complete baseline or medical history in a single EHR database.

We observed low EHR data completeness in this study. Although medical records were collected and processed for NHI reimbursements, EHR and claims data serve different purposes for healthcare organization administration. EHR databases often accommodate more transactions and changes than claims as they reflect the complexity and dynamics of clinical practice. The diagnoses in the EHRs can be re-coded to meet the reimbursement payment schemes. On the other hand, only three outpatient or five inpatient diagnosis codes are available in the NHI claims data; diagnosis codes that were placed at a lower position in the EHRs might not be recorded in the claims.

We faced considerable sample loss when creating a patient cohort with continuous care using the EHR databases studied. Requiring 6-month baseline and 24-month follow-up continuous data left only 10% of the initial sample size in the NTUH-iMD and the CGRD and 42% of the initial sample size in the NCKUH EHR. This suggests that an EHR-based study of diabetes might suffer from issues such as left-censoring, loss to follow-up, or out-of-system care. Ideally, patients should only visit one healthcare delivery system for severe or complicated diseases under the shared-care and dual-referral system in Taiwan. In reality, however, patients can easily choose different healthcare providers and visit a specialist without referral under the NHI. The NTUH and CGMH are medical centers located in the metropolitan areas with a high density of medical institutions, while the NCKUH is located in a city with a lower density of medical institutions. With low geographic or financial burden, patients with multiple diseases are likely to go to different healthcare delivery systems for different conditions, which may be reflected in our finding of low data continuity recorded in the NTUH-iMD and CGRD. Given the high sample attrition and considerable variation in sample loss across healthcare delivery systems, it is important for studies to assess the continuity of patient care when conducting EHR-based research for long-term outcomes.

A trade-off of high EHR data continuity and completeness is potential selection bias and may affect the generalizability of study results (Sun et al., 2022). As EHR databases are generated when patients enter the healthcare system, they reflect not only patients’ health status but also the interaction between the patients and the healthcare systems (Verheij et al., 2018). Patients with a high level of EHR data continuity are generally those with a higher number of encounters. These patients are often sicker or more medically adherent and may not represent the general patient population. The frequency of visits or tests could be affected by insurance coverage or reimbursement policy. This may be less of an issue in this study as Taiwan has universal coverage. However, caution should be exercised when comparing EHR data over time or across different healthcare systems.

This study had some limitations. First, the differences in clinical practices regarding disease coding and treatment may lead to data variation across the three EHR systems. Second, this study only focused on OHA users, so the results may not be generalizable to other patient populations. Third, due to the data governance policies, we could only link one EHR database, the NTUH-iMD, to the claims data. Further evaluations of EHR data completeness in different delivery systems are necessary. Third, although claims databases capture medically attended events that occur across multiple delivery systems, only three outpatient or five inpatient diagnosis codes are available in the NHIRD, limiting its ability to capture all diagnoses. Finally, the EHR data assessed in this study were from 2011 to 2016, which covered the initial period of coding transition from ICD-9-CM to ICD-10-CM. Further studies may evaluate EHR data completeness with more updated data.

In conclusion, EHR data continuity for patients receiving OHAs was relatively low and varied across healthcare delivery systems. The degree of out-of-system care may affect the appropriate length of study follow-up, as well as data completeness of outcomes and baseline comorbidities. The study results suggest that careful evaluation of data completeness is necessary to ensure the validity of real-world evidence generated by EHR data.

## Data Availability

The data analyzed in this study is subject to the following licenses/restrictions: Due to the sensitive nature of the data collected for this study, requests to access the dataset from qualified researchers trained in human subject confidentiality protocols may be sent to the Health and Welfare Data Science Center, Ministry of Health and Welfare of Taiwan at stdlwu@mohw.gov.tw. Data from the Integrated Medical Database of National Taiwan University Hospital (NTUH-iMD), the Chang Gung Research Database (CGRD) from Chang Gung Memorial Hospitals (CGMHs), and the EHR of National Cheng Kung University Hospital (NCKUH) are not available for access outside of the facility.
