# Supplementary material for: Continuity and Completeness of Electronic Health Record Data for Patients Treated With Oral Hypoglycemic Agents: Findings From Healthcare Delivery Systems in Taiwan
Source: Front Pharmacol. 2022 Apr 4;13:845949. doi: 10.3389/fphar.2022.845949 (PMC9015706; doi:10.3389/fphar.2022.845949)
Supplement: Supplementary file 1 [file DataSheet1.docx]

**SUPPLEMENTARY FILES**

**Table S1**. Drug codes used to identify oral hypoglycemic agents

| ATC code | Description |
| --- | --- |
| A10BA | biguanides |
| A10BB | sulfonylureas |
| A10BC | sulfonamides (heterocyclic) |
| A10BD | combinations of oral blood glucose lowering drugs |
| A10BH | dipeptidyl peptidase 4 (DPP-4i) inhibitors |
| A10BJ | glucagon-like peptide-1 (GLP-1) analogues |
| A10BK | sodium-glucose co-transporter 2 (SGLT2) inhibitors |
| A10BX | other blood glucose lowering drugs, excl. insulins |

ATC, anatomical therapeutic chemical.

**Table S2**. Diagnosis codes used in the study to define study cohort and select outcomes.

| Condition | ICD-9-CM | ICD-10-CM |
| --- | --- | --- |
| ELIGIBILITY |  |  |
| Type 2 diabetes mellitus | 250.00, 250.02, 250.10, 250.12, 250.20, 250.22, 250.30, 250.32, 250.40, 250.42, 250.50, 250.52, 250.60, 250.62, 250.70, 250.72, 250.80, 250.82, 250.90, 250.92 | E11 |
| OUTCOMES |  |  |
| Cardiovascular diseases |  |  |
| Acute myocardial infarction | 410.xx | I2101, I2102, I2109, I2111, I2119, I2121, I2129, I213, I214, I220-I222, I228, I229, R0989 |
| Stroke | 430-437, V12.54 | G450-G452, G454, G458-G468, I6000-I6002, I6010-I6012, I6020-I6022, I6030-I6032, I604, I6050-I6052, I606-I619, I6200-I6203, I621, I629, I6300, I63011, I63012, I63019, I6302, I63031, I63032, I63039, I6309, I6310, I63111, I63112, I63119, I6312, I63131, I63132, I63139, I6319, I6320, I63211, I63212, I63219, I6322, I63231, I63232, I63239, I6329, I6330, I63311, I63312, I63319, I63321, I63322, I63329, I63331, I63332, I63339, I63341, I63342, I63349, I6339, I6340, I63411, I63412, I63419, I63421, I63422, I63429, I63431, I63432, I63439, I63441, I63442, I63449, I6349, I6350, I63511, I63512, I63519, I63521, I63522, I63529, I63531, I63532, I63539, I63541, I63542, I63549, I6359, I636, I638, I639, I6501-I6503, I6509, I651, I6521-I6523, I6529, I658, I659, I6601-I6603, I6609, I6611-I6613, I6619, I6621-I6623, I6629, I663, I668-I672, I674-I680, I682, I688 |
| Nephropathy |  |  |
| Diabetic nephropathy | 250.4x | E1021, E1022, E1029, E1065, E1121, E1122, E1129, E1165 |
| Acute glomerulonephritis | 580.xx | N000-N019, N08 |
| Nephrotic syndrome | 581.xx | N020-N029, N040, N041-N049, N08 |
| Chronic glomerulonephritis | 582.xx | N030-N039, N08 |
| Nephritis/nephropathy | 583.xx | E1021, E1121, N050-N079, N08, N140-N144, N150, N158, N159, N16, N171, N172 |
| Acute kidney failure | 584.xx | N170-N172, N178, N179 |
| Chronic renal failure | 585.xx | N184,-N186, N189 |
| Renal failure NOS | 586.xx | N19 |
| Renal sclerosis, unspecified | 587.xx | N261, N269 |
| Disorders resulting from impaired renal function | 588.xx | N250, N251, N2581, N2589, N259 |
| Unspecified disorder of kidney and ureter | 593.xx | N134, N135, N1370, N1371, N13721, N13722, N13729, N13731, N13732, N13739, N138, N139, N181-N183, N189, N280, N281, N2881-N2884, N2886, N2889, N289, N29, R802 |
| Hypertensive chronic kidney disease | 403.xx | I120, I129 |
| Hypertensive heart and chronic kidney disease | 404.xx | I130, I1310, I1311, I132 |
| Encounter for extracorporeal dialysis | V56.xx | Z4901, Z4902, Z4931, Z4932 |
| Proteinuria | 791.0 | R800, R801, R803, R808, R809 |
| Kidney replaced by transplant | V42.0 | Z940 |
| Phlebitis and thrombophlebitis | V45.1 | Z992 |
| Personal history of nephrotic syndrome | V13.03 | (NA) |
| Heart failure | 428.xx | I501, I5020-I5023, I5030-I5033, I5040-I5043, I509 |

ICD-9-CM, International Classification of Diseases, Ninth Revision, Clinical Modification; ICD-10-CM, International Classification of Diseases, Tenth Revision, Clinical Modification

**Table S3.** Definitions of EHR-claims outcome agreement and EHR outcome representativeness

| Outcomes presented in | | Claims | |
| --- | --- | --- | --- |
|  |  | Yes | No |
| Electronic Health Records (EHRs) | Yes | a | b |
|  | No | c | d |

$$EHR-Claims Outcome Agreement =\frac{a+d}{a+b+c+d}$$

$$EHR Outcome Representativenss=\frac{a}{a+c}$$
